# Role of Ubiquitin Ligases and Conjugases in Targeted Cancer Therapy

**DOI:** 10.3390/cancers15133460

**Published:** 2023-07-01

**Authors:** Jerry Vriend

**Affiliations:** Department of Human Anatomy and Cell Science, Rady Faculty of Health Sciences, University of Manitoba, Winnipeg, MB R3E 0J9, Canada; jerry.vriend@umanitoba.ca

The ubiquitin proteasome system regulates the activity of many short-lived proteins in cells. Ubiquitin is added to proteins through a three-step process sequentially involving ubiquitin-activating enzymes (E1), ubiquitin-conjugating enzymes (E2), and ubiquitin ligases (E3). Ubiquitin may be removed from proteins, and ubiquitin chains edited by deubiquitinases, as needed. While initial studies related the UPS to its role in the degradation of damaged or excess proteins [1], considerable progress has been made in determining the role of the ubiquitin proteasome pathway in many cellular processes, including transcription, cell cycle regulation, cell membrane receptor regulation, immune response, and autophagy. Ubiquitin ligases and conjugases as well as deubiquitinases may also act to promote or suppress tumor initiation, maintenance, and growth.

In this Special Issue, we present original research and reviews on the role of ubiquitinaton in the transformation, invasiveness, proliferation, and maintenance of various cancer cells. Various components of the UPS have been shown to have some role in a variety of cancers. While regulation of the cell cycle may be a common theme relating the ubiquitin proteasome pathway to many cancers, additional themes relating the UPS to cancer include control of apoptosis, epigenetics, control of immunity and the immunoproteasome, and regulation of DNA damage repair. From a practical point of view, ubiquitin ligases and their adaptors provide a large number of therapeutic targets, most of which remain unidentified. The challenge is to identify them and to engineer small specific molecular inhibitors that, alone or in conjunction with chemotherapeutic agents, improve the outcome of cancers without serious side effects. Bioinformatics programs are useful in providing information on the role of the ubiquitin proteasome system in various cancers [2,3,4]. The gene encoding the ubiquitin conjugase UBE2C has been described as overexpressed in most cancers [5,6,7]. This can be verified through publicly available microarray datasets. In microarray datasets, UBE2C expression is overexpressed in glioblastoma (Sun dataset [8], in some types of medulloblastoma [9,10], in high-grade serous ovarian cancer [11], and in triple-negative breast cancer [12]. UBE2C is a regulator of cell cycle phase progression and, as such, should be investigated further as a potential therapeutic target. UBE2T, another ubiquitin conjugase, contributes to DNA repair [13], along with BRCA1. BRCA1 is well known for its mutations’ relationship with breast cancer risk. BRCA1 is less well known as a ubiquitin ligase [14].

Original reports in this Special Issue contribute information concerning the interaction of ubiquitin ligases and cancer. The regulation of the transcriptional factor KAP1, also known as TRIM28 (a ubiquitin ligase), by SMURF2, another ubiquitin ligase, in breast cancer cells was shown by Shah et al. [15]. Mutations of substrates binding motifs of VHL, a ubiquitin ligase adaptor that functions as an E3 substrate recognition protein, were reported by Faclconieri et al. and found to be associated with VHL tumors [16]. The discovery of an inhibitor of the deubiquitinase OTUD7B by Chen and colleagues provides evidence for a potential therapeutic target in leukemia cells [17].

The report of Chang et al. showed that the ubiquitin ligase adaptors FBXL8 and FZR1 (which is a regulator of the APC/c cell cycle complex) were potential therapeutic targets in breast cancer [18]. FZR1 (also known as CDH1) and CDC20 interact with the APC/c ubiquitin ligase complex at different stages of the cell cycle [19]. 

The reviews in this Special Issue deal with ubiquitination and cervical cancer, endometrial cancer, and ovarian cancer. One review deals with the role of ubiquitin ligases in the regulation of autophagy as it relates to cancers.

The p53 tumor suppressor gene has been implicated in various cancers including endometrial and cervical cancer. It is regulated by an oncogenic protein, MDM2, which is also an E3 ligase. The reported mutation rate of the E3 ligase MDM2 is greater than 10% in endometrial cancer, while the reported mutation rate of the E3 ligase adaptor FBXW7 is greater than 15% in this cancer. These data suggest that mutations or loss of function of E3 ligase complexes may be a significant cause of cancer. 

Increased transcription and translation of E3 ligase components may also lead to cancers. In some cancers, the neddylation inhibitor MLN4924 (Pevonedistat) has been shown to be useful [20,21,22]. It has been suggested as a potential therapeutic agent in endometrial carcinoma, in cervical cancer, and in ovarian cancer [23,24]. Small molecular inhibitors of specific ubiquitin ligases or their substrate adaptors could be therapeutically useful once the role of the ubiquitin ligase complex in a particular cancer has been established. 

In high-grade serous ovarian cancer, transcription of genes encoding the ubiquitin conjugases UBE2C and UBE2T is increased several fold (5.54- and 4.85-fold, respectively) compared to transcription in low-malignant-potential ovarian cancers [23]. An inhibitor that would bring these levels back to normal physiological levels could be useful in reducing proliferation in ovarian cancers, as well as in other cancers with elevated UBE2C and UBE2T transcription and translation. A substantial amount of research would be required to determine whether it would be possible to produce inhibitors of these conjugases that are therapeutically effective and not overly toxic to normal cells. 

PROTAC techniques make use of ubiquitin ligases to target proteins of interest. A PROTAC consists of a molecule that binds the target of interest, a molecule that binds to an E3 ligase, and a linker molecule. The PROTAC uses the cell’s own E3 ubiquitin ligase complex to ubiquitinate the target protein of interest; the E3 ligase complex can be recycled numerous times after proteasomal degradation of a protein of interest. PROTAC technology has the potential to target proteins that are specifically essential to a particular tumor with minimal or no damage to the essential functions of non-tumor cells [23,25]. PROTACs targeting androgen and estrogen receptors have entered phase II clinical trials for prostate cancer and breast cancer (Arvinas, Inc., New Haven, CY, USA) [26], while several others have entered phase I clinical trials. Recently, progress in the use of PROTACs for the treatment of lung cancer has been reported by Li and colleagues [27]. PROTAC technology thus has great potential for the treatment of cancers in which the viability of the cancer depends on specific proteins that can be targeted.
